# Parent-of-origin effects on nuclear chromatin organization and behavior in a Drosophila model for Williams–Beuren Syndrome

**DOI:** 10.18699/VJ21.054

**Published:** 2021-09

**Authors:** A.V. Medvedeva, E.V. Tokmatcheva, A.N. Kaminskaya, S.A. Vasileva, E.A Nikitina, S.A. Zhuravlev, G.A. Zakharov, O.G. Zatsepina, E.V. Savvateeva-Popova

**Affiliations:** Pavlov Institute of Physiology of the Russian Academy of Sciences, St. Petersburg, Russia; Pavlov Institute of Physiology of the Russian Academy of Sciences, St. Petersburg, Russia; Pavlov Institute of Physiology of the Russian Academy of Sciences, St. Petersburg, Russia Institute of Bioorganic Chemistry of the Russian Academy of Sciences, Moscow, Russia; Pavlov Institute of Physiology of the Russian Academy of Sciences, St. Petersburg, Russia; Pavlov Institute of Physiology of the Russian Academy of Sciences, St. Petersburg, Russia Herzen State Pedagogical University of Russia, St. Petersburg, Russia; Pavlov Institute of Physiology of the Russian Academy of Sciences, St. Petersburg, Russia; Pavlov Institute of Physiology of the Russian Academy of Sciences, St. Petersburg, Russia; Engelhardt Institute of Molecular Biology of the Russian Academy of Sciences, Moscow, Russia; Pavlov Institute of Physiology of the Russian Academy of Sciences, St. Petersburg, Russia

**Keywords:** POE (parent-of-origin effects), 3D nuclear architecture, chromatin ectopic contacts, LIM-kinase 1 (LIMK1), actin, mir-RNA, learning acquisition, memory formation, locomotion, эффект родительского происхождения аллелей, 3D организация ядра, эктопические контакты хромосом, LIM-киназа 1 (LIMK1), актин, микро-РНК, обучение; формирование памяти, локомоция

## Abstract

Prognosis of neuropsychiatric disorders in progeny requires consideration of individual (1) parent-of-origin effects (POEs) relying on (2) the nerve cell nuclear 3D chromatin architecture and (3) impact of parent-specific miRNAs. Additionally, the shaping of cognitive phenotypes in parents depends on both learning acquisition and forgetting, or memory erasure. These processes are independent and controlled by different signal cascades: the first is cAMPdependent, the second relies on actin remodeling by small GTPase Rac1 – LIMK1 (LIM-kinase 1). Simple experimental model systems such as Drosophila help probe the causes and consequences leading to human neurocognitive pathologies. Recently, we have developed a Drosophila model for Williams–Beuren Syndrome (WBS): a mutant *agn^ts3^* of the *agnostic* locus (X:11AB) harboring the *dlimk1* gene. The *agn^ts3^* mutation drastically increases the frequency of ectopic contacts (FEC) in specific regions of intercalary heterochromatin, suppresses learning/memory and affects locomotion. As is shown in this study, the polytene X chromosome bands in reciprocal hybrids between *agn^ts3^* and the wild type strain **Berlin** are heterogeneous in modes of FEC regulation depending either on maternal or paternal gene origin. Bioinformatic analysis reveals that FEC between X:11AB and the other X chromosome bands correlates with the occurrence of short (~30 bp) identical DNA fragments partly homologous to Drosophila 372-bp satellite DNA repeat. Although learning acquisition in a conditioned courtship suppression paradigm is similar in hybrids, the middle-term memory formation shows patroclinic inheritance. Seemingly, this depends on changes in miR-974 expression. Several parameters of locomotion demonstrate heterosis. Our data indicate that the *agn^ts3^* locus is capable of trans-regulating gene activity via POEs on the chromatin nuclear organization, thereby affecting behavior.

## Abstract (ru)

Прогноз развития нейропсихиатрических заболеваний требует учитывать родительское проис- хождение аллелей как существенный фактор предрасположенности у потомства. Родительское наследование определяет 3D организацию хромосом в ядре нервных клеток, в том числе за счет эпигенетического влияния микро-РНК генеративных клеток родителей. Кроме того, когнитивные нейропатологии у родителей зависят от двух процессов – обучения и забывания, или стирания памяти. Эти процессы независимы и контролируются раз- ными сигнальными каскадами: обучение – цАМФ-зависимым, забывание – каскадом ремоделирования актина: малая ГТФаза Rac1 – LIMK1 (LIM-kinase 1). Для понимания становления нейропатологии человека необходимо привлечение простых модельных объектов. Нами создана модель синдрома Вильямса–Бойерна на дрозофи- ле с мутационным повреждением гена *dlimk1* – *agnostic* (*agn^ts3^*), кодирующего ключевой фермент ремодели- рования актина LIMK1. У *agn^ts3^* повышена частота формирования негомологичных контактов в специфических районах интеркалярного гетерохроматина, резко нарушены способность к обучению, формированию памяти и локомоция. У реципрокных гибридов между *agn^ts3^* и линией дикого типа **Berlin** частота эктопических контактов, сформированных дисками политенных хромосом, зависит от направления скрещивания, воспроизводя либо отцовские, либо материнские свойства. Биоинформационный анализ показывает, что частота эктопических контактов между X:11AB и другими районами Х хромосомы обусловлена присутствием короткого (~30 п. н.) фрагмента ДНК, частично гомологичного участку 372 п. н. сателлитной ДНК. Гибриды, имея одинаковую спо- собность к обучению в парадигме условно-рефлекторного подавления ухаживания, проявляют патроклинный характер наследования среднесрочной памяти. Это может быть связано с уровнем экспрессии миР-794. Параметры локомоторной активности проявляют гетерозис. По-видимому, локус *agn^ts3^* осуществляет трансрегуля- цию пространственной организации ядра, тем самым влияя на количественные признаки (поведение).

## Introduction

Genome plasticity is ensured by the architecture of specific nuclear loci and nuclear localization of transcriptional machinery (Medrano-Fernández, Barco, 2016; Iourov et al., 2019) where the chromatin organization is the priority-driven factor (Ito et al., 2014; Medrano-Fernández, Barco, 2016; Li et al., 2018). The outcome of recent achievements in systems biology is the notion that the plasticity of 3D chromatin architecture of nervous cell nuclei plays the leading role in cognition and neuropsychiatric disorders (Medrano-Fernández, Barco, 2016; Kim et al., 2018; Iourov et al., 2019). The epigenetic component is still an underestimated source of psychomotor disturbances and neuronal diversity (Savvateeva-Popova et al., 2017). Therefore, a new field of human biomedical research named molecular cytogenetics and cytogenomics (Iourov et al., 2008), or chromosomics (Liehr, 2019) has evolved. The main goal of chromosomics is the study of chromosomes, their 3D architecture in the interphase nucleus, the outcomes of chromosomal sub-region plasticity and gene interactions for shaping interindividual and intercellular genomic variations in normal behavior and disease.

Recently, this topical problem has turned out to be the pursuit of understanding the concomitant role of active forgetting, since the antithesis to learning acquisition is the forgetting or memory erasure (Davis, Zhong, 2017). Both processes are independent and controlled by different signal cascades: learning acquisition and memory consolidation occurs via cAMP cascade, its components being CREB and C/EBP. Active forgetting relies on actin remodeling cascade responsible for structural alterations of neurons and synapses: small GTPase Rac1 – LIMK1 (the key enzyme of actin remodeling LIM-kinase 1) and its phosphorylation substrate сofilin. The absence of Rac1-dependent forgetting causes the autistic spectrum disorders. Expression changes (active or non-active state) of LIMK1 and cofilin lead to different neuro pathologies. The most studied example embracing all the aforementioned facets of manifestations is Microdeletion (Deletion) Williams–Beuren Syndrome, or WBS in 7q11.23. WBS deletion leads to cardiovascular pathology, cognitive deficit in visuospatial construction and hypersociability (Kaiser-Rogers, Rao, 2005; Nikitina et al., 2014c). This is because long-term synaptic plasticity and depression determine successfulness of learning and memory, as well as of locomotor behavior, and depend on epigenetic regulation of LIM-kinase 1 (LIMK1) gene, one of approximately 28 genes uncovered by WBS deletion. Epigenetic regulation of LIMK1 activity involves DNA methylation, chromatin remodeling and the noncoding RNA-mediated process (Smrt, Zhao, 2010).

LIMK1, a member of serine/threonine (Ser/Thr) family kinases regulated by the Rho-GTPase pathway, is the key enzyme of actin remodeling cascade. Dendritic spines are actin-rich structures, and spine dynamics is driven mainly by actin remodeling, thus sharing several molecular pathways with dendrite growth (Smrt, Zhao, 2010). Increasing sets of evidence suggest that nuclear actin also plays a pivotal role in transcriptional regulation and DNA repair. Interestingly, monomeric actin is a stoichiometric subunit of a variety of chromatin remodeling complexes. Ashift between monomeric and polymeric states modifies activity of histone deacetylases (Klages-Mundt et al., 2018).

Recently, we have developed a simple and appropriate Drosophila model for chromosomics (Savvateeva-Popova et al., 2017) using the properties of the *agnostic* locus harboring LIMK1 gene (X:11AB). This region possesses the properties of intercalary heterochromatin. Being a hotspot of chromosome breaks, ectopic contacts, underreplication and recombination, the region attains strain-specific architecture marked by single base changes and small insertion/deletions. The EMS-induced temperature-sensitive (ts) mutation *agn^ts3^* carries the insertion of transposable element (TE) from Tc1/mariner superfamily ~460 bp downstream 3′UTR of Drosophila LIMK1 gene (*dlimk1*), as well as A/T-rich 28 bp insertion within intron 1 of *dlimk1* capable of pairing with 5′ TIR of the TE.

When maintained at 29 °C, *agn^ts3^* shows a temperaturesensitive lethality at all stages of development except for the imaginal stage. At normal temperature, the adult flies show drastic learning acquisition and memory retention defects, as well as locomotor impairments and amyloid-like inclusions (Nikitina et al., 2014b; Kaminskaya et al., 2015). Stress exposure (heat shock for 30 min at 37 °C) suppresses these manifestations (Nikitina et al., 2012, 2014a). Also, *agn^ts3^*mutation leads to: (1) LIMK1 and p-cofilin increase in the adult brain and salivary glands of 3rd instar larvae at 22–25 °C and a fall down to the level of the wild type strain *Canton S* at 29–37 °C; (2) high level of ts-induced recombination within *agn^ts3^* region; (3) 3-fold increase in frequency of non-allelic ectopic contacts (FEC) within 2L arm of the chromosome 2 and in the 11В X chromosome region (Medvedeva et al., 2010). Additionally, miRNAs expression including the biomarkers for human neuropathologies is drastically reduced in *agn^ts3^* relative to the wild type strains (Savvateeva-Popova et al., 2017).

**Fig. 1.**
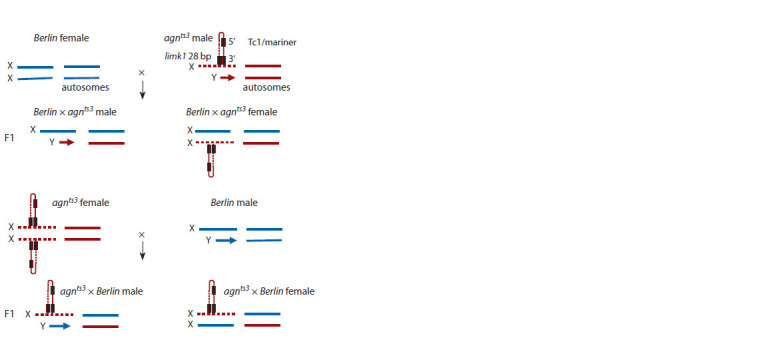
Architecture of Drosophila chromosomes in the studied strains.

*agn^ts3^*-specific nuclear organization is shaped in early embryogenesis alongside with formation of chromosomal heterochromatin regions. Intrinsic *agn^ts3^* FEC is maternally inherited (Medvedeva et al., 2010). Therefore, *agn^ts3^* is a promising model for studies on parent-of-origin effects (POEs) on progeny considered as significant causative factors of psychiatric disorders (Zayats et al., 2015). For instance, a 1.5 Mb WBS deletion recurrently arises *de novo* and depends on POEs: maternal origin leads to more severe developmental abnormalities and microcephaly (Pérez Jurado et al., 1996). Moreover, when WBS deletion has a paternal origin, expression levels of a number of genes within the WBS deletion decrease. Among these genes crucial for the brain development is a gene for general transcription factor II-I (GTF2I). It regulates transcription by binding to DNA and histone deacetylase (HDAC) (Collette et al., 2009). The main goal of the study is the analysis of POEs role in shaping quantitative traits, namely learning acquisition, memory retention, locomotion, and miRNAs expression while using the advantages of the Drosophila model for POEs in progeny from reciprocal crosses between *agn^ts3^* and the wild type strain **Berlin** (Fig. 1).

To meet the requirements of chromosomics, FECs between the region X:11AB and the other bands of the X chromosome may be estimated as an indicator of chromosomal spatial organization. This approach is justified by the existence of late-replicating genomic territories including the underreplicated regions of polytene chromosomes. These regions overlap with late-replicating regions of mitotically dividing cells (Belyakin et al., 2005). Suppression of SUUR gene responsible for underreplication of intercalary heterochromatin and, as a consequence, for the ectopic pairing leads to death in early embryogenesis (Belyaeva et al., 1998). Additionally, to elucidate the contribution of DNA sequence homology as components of epigenetic regulation in the ectopic chromatin pairing we have developed the special software package Homology Segment Analysis.

## Materials and methods

**Drosophila stocks.** The fly stocks used belong to Biocollection of Pavlov Institute of Physiology of the Russian Academy of Sciences: • *Berlin*, a wild type strain; • *Canton S*, a wild type strain; • *agn^ts3^*, a temperature-sensitive mutation on *Canton S* genetic background within *agnostic* locus (X:11AB) affecting *dlimk1* activity.

The reciprocal hybrids between *agn^ts3^* and *Berlin* were used because *Berlin* *dlimk1* sequence is closer to FlyBase reference sequence (Savvateeva-Popova et al., 2017). At the same time, the reciprocal hybrids *agn^ts3^*×Canton S (the genetic background for *agn^ts3^*) and Canton S×*agn^ts3^* demonstrate exactly the same cognitive behavior as reciprocal hybrids with *Berlin* (Vasiljeva et al., 2019) approving the usage of *Berlin*.

Figure 1 shows the X chromosome and autosomes architecture in *Berlin* and *agn^ts3^* female and male parents and F1 female and male progeny from reciprocal crosses with the accent on the putative hairpin formed in the X chromosome by 28 bp A/T rich insertion within intron 1 of *dlimk1* gene and 3′ end of Tc1/mariner element.

Flies were maintained on standard Drosophila yeast-raisin medium at +22 ± 0.5 °С under a 12-h light/dark cycle. For the memory and locomotion tests, males were collected upon eclosion without narcotization and kept individually in culture vials till the behavioral experiments on the 5th day

**Fig. 2.**
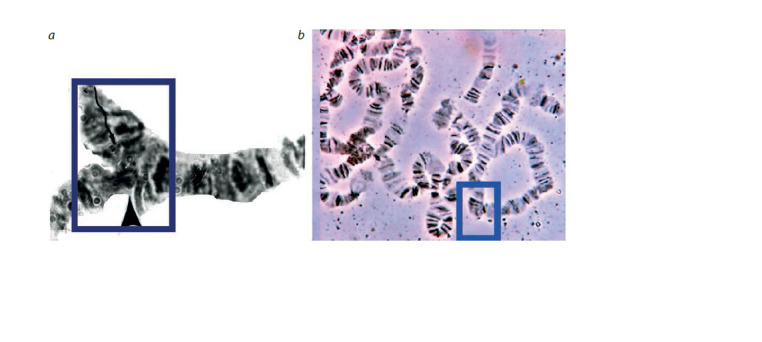
Examples of ectopic contacts in the 2L (a) and in the X (b) chromosomes.

**Estimation of frequency of ectopic contacts (FECs).** The aceto-orcein squash preparations were prepared from salivary glands of III instar D. melanogaster female larvae. 20 to 30 animals were examined, therefore the number of analyzed chromosomes varied from 300 to 500. The examples of ectopic contacts are presented on Fig. 2. The number of non-homologous contacts between the region X:11AB and different bands of the X chromosome was calculated and expressed as per cent of the total number of the examined nuclei. FECs in parents and F1 reciprocal hybrids were compared using Student’s t-test. Identification of genes localized in the X chromosome bands forming contacts with the 11AB region was performed using NCBI Genome Data Viewer database https://www.ncbi.nlm.nih.gov/genome/gdv/ and molecular function of identified genes was derived from FlyBase https://flybase.org

** analysis of DNA segments homology.** D. melanogaster genome sequence (release 6) was taken from (Zerbino et al., 2018). Special software package Homology Segment Analysis searching the matches of short singlestranded DNA fragments within the chromosome areas involved in the ectopic pairing in Drosophila has been developed. The software written in Python 3 can be freely downloaded from (Zhuravlev, 2019a). Software version from git (commit 41719cddc6283edbd79c5bf2aee237cde48d4b7d) was used. The algorithm of the program is described in brief in (Zhuravlev, 2019b). The exact run parameters for 11AB region are following: segmentanalysis.py -v -s 30 dm6.nounmapped. fa.gz :X:11982050:12772070 DmelMapTable.160615c.bed

Here, dm6.nounmapped.fa.gz is Drosophila genome canonical sequence (Ensembl release 6) and DmelMapTable. 160615c.bed is chromosome bands location from Ensembl database converted to BED format. Both files are included in software repository.

For other tested regions, all parameters were the same except for the regions location.

**Preparation of miRNAs libraries and bioinformatic analysis.** The detailed description of the procedure is given in (Savvateeva-Popova et al., 2017). Extract RNA reagent (Evrogen, Russia) was used for total RNA extraction from adult 5 days old males. To obtain the fraction of small RNA, 25 μg of total RNA were separated using 15 % polyacrylamide gel electrophoresis in the presence of Urea (8 M) following excision of small RNA fraction corresponding to 21–29 nts. Illumina TruSeq Small RNA prep kit (Illumina, USA) was used for small RNA libraries preparation. Sequencing was performed on an Illumina HiSeq 2000 platform.

The amount of mapped miRNAs reads was counted by BEDTools (v. 2.22) and mirbase annotation (r. 19) (Quinlan, Hall, 2010). Analysis of differentially expressed miRNAs was performed using edgeR (v. 3.10.2) package in R environment (v. 3.2.2) (Robinson et al., 2010). miRNA functions were derived from miRBase (Kozomara et al., 2019). Small RNA libraries were deposited in NCBI SRA under the number PRJNA633483.

**Locomotor activity.** Computer-aided automatic device for simultaneous registration of 20 animals is described in (Zakharov et al., 2012). The experiment lasted for 1 h. Spontaneous locomotor activity of flies was detected in a plate with eight chambers and transparent cover using high-resolution video camera. Software used for locomotor activity analysis is freely available at (Zakharov, 2017).

The following parameters of locomotion were assessed: activity index (%); run frequency (the number of run bouts in 100 seconds); running speed (mm/s). The full record was divided into 1 s quanta, and the mean speed of fly movement in each quantum was calculated. If the result was less than the threshold value (5 mm/s), the fly was considered to be resting during this time quantum; otherwise, it was considered moving. Neighboring quanta with similar movement pattern were merged in intervals of moving and resting. Activity index is determined as a time spent in movement. Running speed is an average fly speed, determined using only intervals of movement. The Kruskal–Wallis analysis of variance with the multiple comparison of mean ranks was used to compare all the experimental groups.

**Learning acquisition and middle-term memory formation in Drosophila males.** Detailed description of learning/ memory assessments in conditioned courtship suppression paradigm (CCSP) and specially designed software for observation and statistical analysis (randomization test) of learning indices based on courtship indices is given in (Kamyshev et al., 1999). CCSP employs the natural stimuli of Drosophila courtship. Both virgin and fertilized females emit an aphrodisiac pheromone, attracting a naïve male without courtship experience. However, a fertilized female rejects a male at the courtship stage of attempted copulation via emitting an aversive pheromone. Repetitive rejections during 30 min training provoke a kind of learned helplessness when a male stops courting another female. This courtship suppression might last for one hour when test female is virgin and for eight hours when fertilized. Males with defective memory formation continue to court after such a training as vigorously, as naïve males.

The courtship index (CI, percentage of time spent in courtship) was calculated for each male. The learning index (LI) was computed according to the formula:

**Formula 1.**
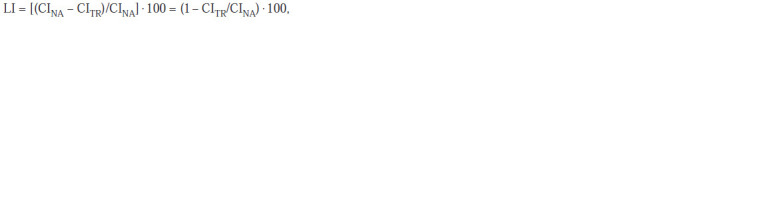
Formula 1 where CINA and CITR are the mean courtship indices for independent samples of naïve and trained males, respectively.

## Results

**Analysis of spatial nuclear organization** delimited to FECs formed by the X chromosome region 11AB

Spatial nuclear organization was analyzed using microscopic images of polytene chromosomes in larvae salivary glands. The results of comparative analysis of FECs between the 11AB region and the other X chromosome regions in *Berlin*, *agn^ts3^* and their hybrids are presented in Table 1. The columns 1–4 show the pattern of FECs between the 11AB region and other X chromosome regions pertinent to listed strains. The absence of significant differences of FECs between columns, i.e. 1 vs 2 and 3 vs 4 indicates matroclinic inheritance, 2 vs 3 indicates hybrid-specific frequencies, 2 vs 4 and 1 vs 3 pinpoints the patroclinic inheritance. The polytene chromosome bands in hybrids demonstrate differences in FECs, a part of them showing either matroclinic properties or properties of the father strain. In certain bands, FECs are similar in reciprocal hybrids (hybrids-specific, i. e. FEC is equal for hybrids, being different from at least one parent) or depend on the direction of a cross, but significantly differ from that of parents.

**Table 1.**
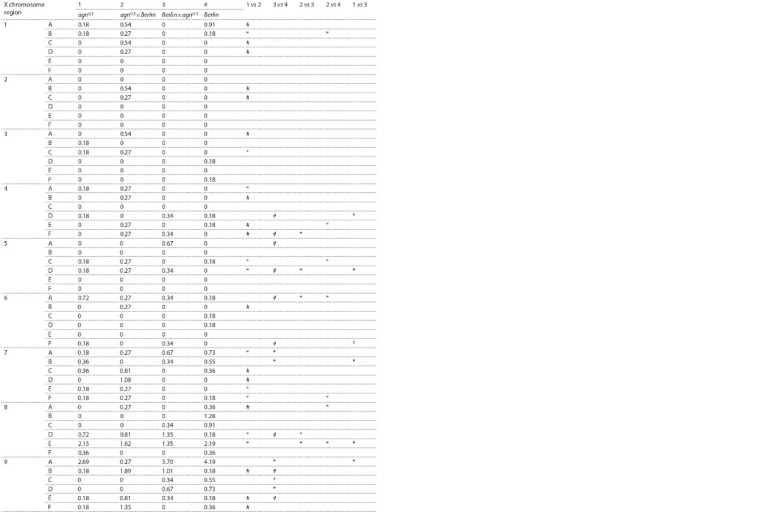
Comparative analysis of X – X:11AB FECs in *Berlin*, *agn^ts3^*, and their reciprocal hybrids

**Table 1(continued):**
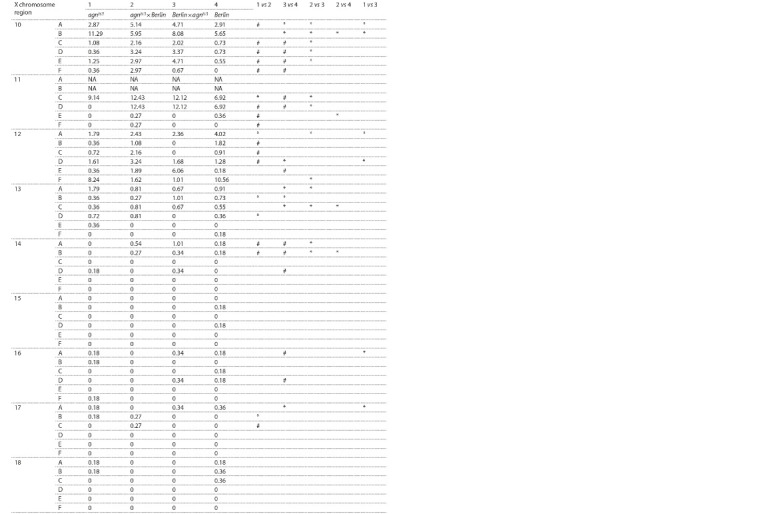
Comparative analysis of X – X:11AB FECs in *Berlin*, *agn^ts3^*, and their reciprocal hybrids

**Table 1(end):**
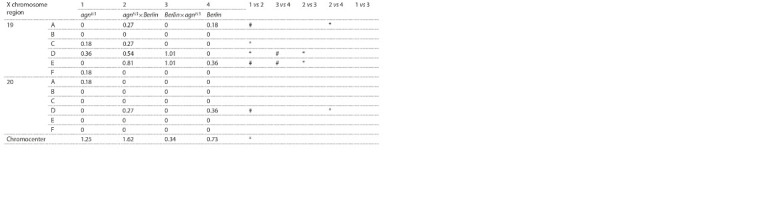
Comparative analysis of X – X:11AB FECs in *Berlin*, *agn^ts3^*, and their reciprocal hybrids Note. NA (not applicable) for 11AB region frequencies indicates that region cannot pair with itself. * No difference, two-sample Student t-test for difference of means (t dif) <1 ref lecting a mode of FEC inheritance: maternal – n columns 1 vs 2 and 3 vs 4, paternal – in columns 2 vs 4 and 1 vs 3, hybrid-specif ic – columns 2 vs 3. # Difference t dif ≥ 1, in columns 1 vs 2 and 3 vs 4 ref lecting the region with FEC higher in hybrids than in parents (heterosis) or regions present only in the hybrid strains. No symbol represents the absence of contacts, therefore statistical signif icance cannot be estimated.

*Berlin*, *agn^ts3^* and their hybrids are presented in Table 1. The columns 1–4 show the pattern of FECs between the 11AB region and other X chromosome regions pertinent to listed strains. The absence of significant differences of FECs between columns, i.e. 1 vs 2 and 3 vs 4 indicates matroclinic inheritance, 2 vs 3 indicates hybrid-specific frequencies, 2 vs 4 and 1 vs 3 pinpoints the patroclinic inheritance. The polytene chromosome bands in hybrids demonstrate differences in FECs, a part of them showing either matroclinic properties or properties of the father strain. In certain bands, FECs are similar in reciprocal hybrids (hybrids-specific, i. e. FEC is equal for hybrids, being different from at least one parent) or depend on the direction of a cross, but significantly differ from that of parents.

**Fig. 3.**
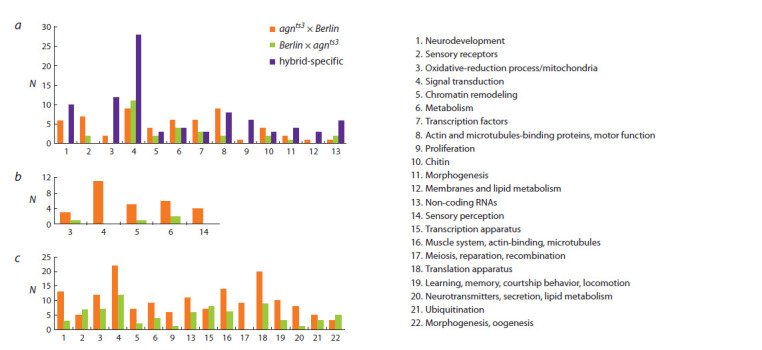
Processes controlled by genes within bands involved in ectopic contacts: a – matroclinic- and reciprocal hybrid-specif ic contacts; b – patroclinicspecif ic contacts; c – hybrids-specif ic contacts. X axis – the biological processes, Y axis – the number of genes.

NCBI Genome Data Viewer software helped to reveal the genes located in the X chromosome bands forming the ectopic contacts. Based on the assumption that shared location of genes determines their functional features (Liu et al., 2019), it was worth elucidating what biological processes are under the influence of epigenetic factors related to allelic parentof- origin. Therefore, the grouping of genes implied their involvement in control of a certain biologic process (Fig. 3).

Figure 3, a presents provisional definition of the biological processes controlled by genes within bands forming contacts with 11AB region either in matroclinic or in reciprocal hybridspecific manner. Among the functional groups of genes having increased number in *agn^ts3^*×Berlin relative to the reciprocal hybrid are genes for motor proteins (8, 4.5-fold) and sensory receptors (2, 3.5-fold). Genes involved in neurodevelopment, oxidative-reduction process and proliferation do not present in Berlin×*agn^ts3^* progeny. Heterosis, when the number of genes involved in hybrid-specific contacts is more than 2-fold higher than in both parents, is manifested for oxidative-reduction process (3), signal transduction (4), proliferation (9) and noncoding RNAs (13).

The biological processes and genes involved in ectopic contacts with 11AB in a mode of father strain are shown in Fig. 3, b. Among them are the genes responsible for chromatin remodeling (5, 5-fold increase in *agn^ts3^*×Berlin relative to the reciprocal hybrid), reactive oxygen species metabolic process (3, 3-fold), and metabolism (6, 3-fold). Sensory perception and signal transduction groups do not present in Berlin×*agn^ts3^*.

Data on biological processes and the number of genes involved in contacts with 11AB region exclusively in hybrids or with FECs prevailing those of parents (FEC heterosis), are shown on Fig. 3, c. The ratio of gene numbers for *agn^ts3^*×Berlin and Berlin×*agn^ts3^* reveals functional significance of other gene groups. The magnitudes of differences may be ranged as follows: lipid metabolism and neurotransmitter secretion (20, 8-fold); metabolism (6, 2.3-fold); cell proliferation (9, 6-fold; as in previously published evidence on *agn^ts3^* (Tokmacheva, 1995)); chromatine remodeling (5, 3.5-fold). Reparation/ recombination group does not present in Berlin×*agn^ts3^*.

**Profiles of the localized fragment frequencies (LFFs)**

Using our specially designed software Homology Segment Analysis, we analyzed correlation between ectopic pairing and distribution of small identical fragments within contacting regions.

**Fig. 4.**
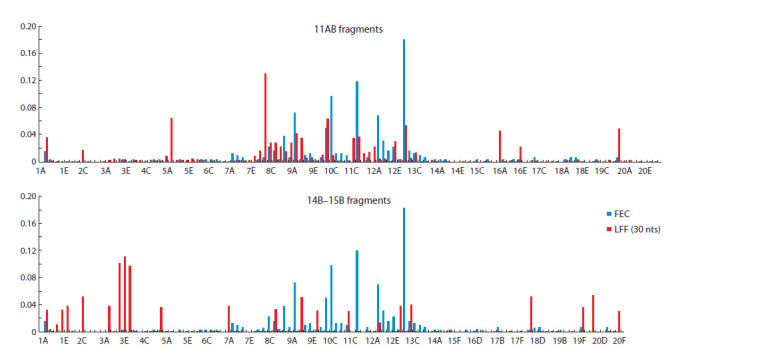
*Berlin* ectopic contacts frequencies (FECs) and localized fragment frequencies (LFFs) for fragments of 11AB and 14B–15B regions. The X chromosome bands are shown.

The region X:11AB (~790 kb) involved in Drosophila X – X:11AB ectopic pairing was selected as the source of small 30 nt fragments, and the profile of X:11AB localized fragment frequencies (LFFs) was constructed for the X chromosome (Fig. 4). The region X:14B–15B was selected as a control region having almost equal length (~790 kb).

Both LFF and the FEC (X – X:11AB) distributions significantly differ from normal. Therefore, we calculated Spearman’s rank-order correlation coefficients (rS) for LFF and the strain-specific FEC (X – X:11AB) (Table 2). A positive correlation between LFF and FEC is observed within the region of interest X:11AB. The influence of the fragment length on rS value has been tested. For 30 nt fragments, rS is highly significant for all Drosophila strains ( p < 0.001). As to LFF (X:14B–15B), there is no significant correlation with FEC (X – X:11AB) ( p ≥ 0.01) pointing to specificity of analysis of DNA homology within the region of ectopic pairing. For 20 nt fragments, there is a false-positive correlation between LFF (X:14B–15B) and *agn^ts3^*×Berlin FEC (X:11AB). Probably, 20 nt fragments are too short to display DNA homology specifically associated with ectopic pairing. The increase in fragments length from 20 to 50 nt leads to decrease in LFF (X:14B–15B) – FEC (X – X:11AB) unspecific correlation and simultaneous increase in LFF – FEC (X:11AB) specific correlation. However, there is a lack of LFF (50 nt) – ECF correlation for *agn^ts3^*. Hence, 30 nt fragment length seems to be optimal in search for the identical fragments within the candidate chromosomal regions involved in ectopic pairing.

**Table 2.**
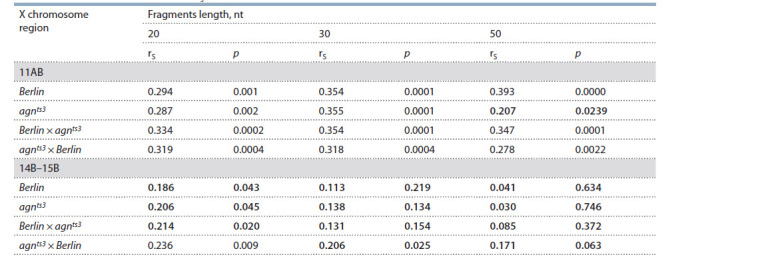
Spearman’s rank correlation (rS) between LFF and strain-specific X – X:11AB FEC Note. Bold: non-signif icant correlation ( p ≥ 0.01).

To find out what DNA sequences impact the ectopic pairing the most, we calculated rS between *Berlin* FEC and the localization frequencies for the several specific X:11AB 30 nt fragments having the highest number of occurrences (NO) in the X chromosome (Table 3). The maximal correlation is evident in the part of 372 bp middle repetitive DNA sequence (marked with asterisk (Waring, Pollack, 1987)). The majority of fragments with significant positive rS values appear to be the parts of a ~50 bp repeat (marked with #) having significant self-complementarity. This sequence also shows an almost complete identity to another part of 372 bp repeat. Such repeats with slight sequence variations occur in both DNA strands of the X chromosome. rS is much lower for (-at-)_15_ repeat and is nearly absent for (-gt-)_15_ (-tc-)_15_ and (-ca-)_15_ repeats, although their NO can be higher. Noteworthy, all r_S_ values for 372 bp repeat fragments are lower compared to r_S_ for the whole set of the localized fragments (see Table 2), hence all of them seemingly impact ectopic pairing.

**Table 3.**
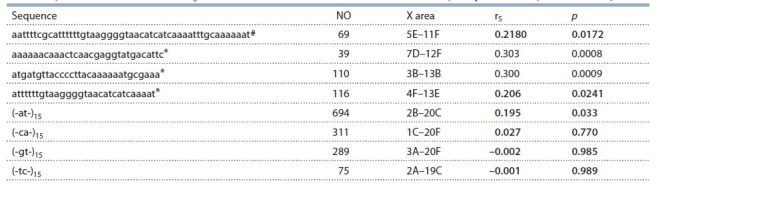
Spearman’s rank correlation (rS) between *Berlin* X – X:11AB FEC and localization frequency values for specific DNA sequences Note. Bold: non-signif icant correlation ( p ≥ 0.01). NO – the number of occurrence of fragment within the X chromosome excluding area 11AB; X area – the area of the X chromosome where the fragment has been localized. # 50 bp fragment; * fragments with a partial homology to 372 bp repeat (see in text).

**miRNAs expression profile**

**Fig. 5.**
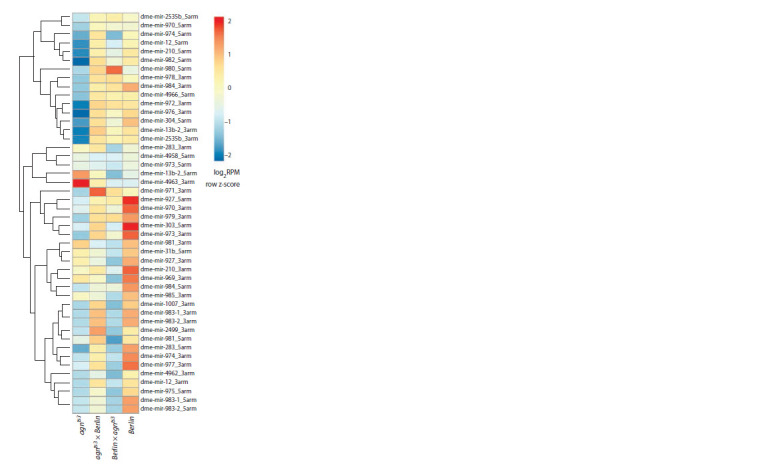
The relative content of miRNAs in reciprocal hybrids compared to parents.

Analysis of miRNAs expression in *Berlin*, *agn^ts3^* and their reciprocal hybrids demonstrates significant differences between males of parent strains and hybrids in content of 44 miRNAs. For the reciprocal hybrids, the heat maps of miRNAs expression are presented in Fig. 5. Among 44 miRNAs 10 miRNAs belong to the same cluster of testis-specific miRNAs (Mohammed et al., 2014). However, only miR-980 and miR-974 are involved in memory processes. Expression of miR-980 suppresses Drosophila memory (Guven-Ozkan et al., 2016), while lowered expression of miR-974 impairs memory formation (Busto et al., 2015)

The heat map represents the RPM-normalized and log2- transformed counts of miRNAs reads with z-scale normalization of the rows. Thirty percent of low-expressed miRNAs were removed from further analysis. Only the miRNAs with altered content in a hybrid compared to at least one parent are shown.

**analysis of parent strains and their reciprocal hybrids**

**Fig. 6.**
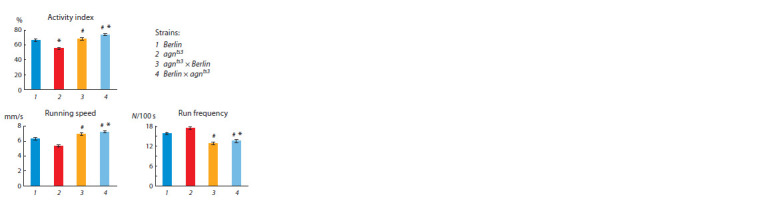
Locomotor activity in *agn^ts3^*, *Berlin* and their reciprocal hybrids. N – the number of run bouts; # the difference from *agn^ts3^*; * the difference from *Berlin* (Kruskal–Wallis analysis, the multiple comparison of ranges, n = 20, p ≤ 0.05).

**Locomotor behavior.** Figure 6 presents the parameters of locomotion. As to activity indices, they are significantly different in *agn^ts3^* and *Berlin*, both hybrids differ from *agn^ts3^* and Berlin×*agn^ts3^* differs from *Berlin*. Running speed and run frequency in *agn^ts3^* and *Berlin* are similar. Both hybrids differ from *agn^ts3^* and Berlin×*agn^ts3^* from *Berlin*. However, the hybrid running speed exceeds that of parents, demonstrating heterosis. Comparatively to parents, run frequency in hybrids is intermediate. At the same time, any alterations in locomotor parameters in hybrids are similar and unidirectional.

**Fig.7.**
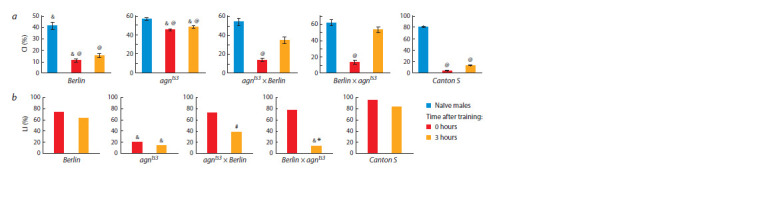
Learning and memory in parents *agn^ts3^*, *Berlin* and their reciprocal hybrids immediately and 3 hrs after training: a – courtship indices; b – learning indices. @ difference from naïve males; # difference from *agn^ts3^*; * difference from *Berlin*; & difference from Canton S (two-way randomization test, p <0.05).

**Learning acquisition and memory formation.**In all strains, CIs of males decrease after training with fertilized females compared to naïve flies (Fig. 7, a). Learning/memory scores in reciprocal hybrids show that memory formation (3 hours after training), but not learning acquisition (0 hours after training), demonstrates patroclinic inheritance (see Fig. 7, b).

When male parent originates from *Berlin* strain in crosses *agn^ts3^*×Berlin, learning and 3-hour memory do not differ from LIs of *Berlin*, but do differ from LIs of *agn^ts3^*. Patroclinic inheritance is evident in the reciprocal hybrid Berlin×*agn^ts3^*. In this case, 3-hour LIs in Berlin×*agn^ts3^* and *agn^ts3^* are similar. The patroclinic inheritance cannot be associated with the Y chromosome, as it is likely to be similar in *Berlin*, *agn^ts3^* and *Canton S* (see Materials and methods, Drosophila stocks description). Also, it cannot be attributed to the X chromosomes, since they are different in *Berlin* and *agn^ts3^*×Berlin. Seemingly, this may be caused by some paternal epigenetic factors, such as miRNAs in cytoplasm of male sperm.

**Discussion**

Studies of genome-wide associations between DNA polymorphisms and phenotypic traits have revealed genetic variants predisposing to different mental diseases. Findings pinpointing the role of POEs in genetic risk for neuropathology open new possibilities for therapy and preventive medicine (Zayats et al., 2015). In this study, we estimated FECs in reciprocal hybrids considering an impact both of genetic variants of *agn^ts3^* gene and epigenetic factors (POEs) in spatial nuclear architecture, learning/memory formation and spontaneous locomotor activity

To exploit the advantages given by the model, we delimited the analysis of spatial nuclear organization to FECs formed by the X chromosome region 11AB harboring *dlimk1* gene. Our assumption was that FEСs partly reflect the restricted homology of short DNA sequences in different, seemingly “non-homologous” regions.

As shown in this study for the short identic fragments within the contacting regions, FECs correlate with LFFs. Although the correlation is rather moderate (~0.35), it is highly specific for 30 nt fragments. Many factors affect ectopic pairing, such as DNA homology, the distance between the interacting regions and epigenetic factors causing the interstrain FECs differences (Zykova et al., 2018). Our computational algorithm concerns only fragments aligned with the X chromosome without gaps. This reveals the partial homology of interacting bands. Thus, the interacting chromosomal areas may be significantly larger than 30 or 50 nts. Although different mechanisms are involved in ectopic pairing, including POEs, our data indicate the significant role of DNA sequence itself. However, as FEC–LFF correlation is mainly observed for specific DNA fragments, their pairing mediated by some proteins or non-coding RNAs cannot be ruled out.

Most of the found 30–50 nt fragments are similar to the D. melanogaster dispersed 372 bp A/T-rich noncoding repeat (Waring, Pollack, 1987). This moderately repeated sequence is located in the euchromatin of the X chromosome between the regions 4 and 14A in ~300–400 copies per haploid genome. The 372 bp repeat is a part of 1.688 g/cm3 class of satellite DNA (1.688X repeats) (Jagannathan et al., 2017). siRNA from the 1.688X repeats is involved in dosage compensation in recognition of the X and autosomal chromatin, thereby delimiting activities of male-specific lethal (MSL) complex to sex chromosomes through up-regulation of the X chromosome (Menon et al., 2014).

The data obtained consider the common mechanisms of ectopic contacts formation and dosage compensations. Seemingly, POEs might influence the spatial chromatin organization, thereby affecting behavioral performances. This indicates that each Drosophila strain possesses its own pattern of ectopic contacts with the region 11AB. The polytene chromosome bands are heterogeneous in their modes of regulation of ectopic pairing. Apart of them is regulated by genes of either maternal, or paternal origin. A separate class is comprised of regions manifesting only hybrid properties. Similar РОЕs were observed for the pattern of methylation and nucleosome distribution within the imprinted loci in humans and plants (Dong et al., 2018; Zink et al., 2018). In both reciprocal crosses, bands 7A, 9A and 13B display the maternal properties. When mother is *agn^ts3^*, FECs in these bands significantly decrease. In this case maternal control of spatial localization and therefore, of gene expression is genetically determined. These are genes controlling membrane receptor regulation (PPYR1) and signal transduction (gce), chromatin remodeling (Top1, HDAC6), axon guidance and chemosensory jumping behavior (acj6 ).

This indicates that each Drosophila strain possesses its own pattern of ectopic contacts with the region 11AB. The polytene chromosome bands are heterogeneous in their modes of regulation of ectopic pairing. Apart of them is regulated by genes of either maternal, or paternal origin. A separate class is comprised of regions manifesting only hybrid properties. Similar РОЕs were observed for the pattern of methylation and nucleosome distribution within the imprinted loci in humans and plants (Dong et al., 2018; Zink et al., 2018).

In both reciprocal crosses, bands 7A, 9A and 13B display the maternal properties. When mother is *agn^ts3^*, FECs in these bands significantly decrease. In this case maternal control of spatial localization and therefore, of gene expression is genetically determined. These are genes controlling membrane receptor regulation (PPYR1) and signal transduction (gce), chromatin remodeling (Top1, HDAC6), axon guidance and chemosensory jumping behavior (acj6 ).

In the cross *agn^ts3^*×Berlin, the number of genes with known functions contacting with 11AB with maternal-specific frequency is 2-fold higher than in reciprocal cross. Possibly, this is due to the *agn^ts3^*-specific miRNAs pattern of expression. The role of miRNAs in maternal inheritance and expression in embryogenesis is sparsely studied. As we have shown earlier, the expression level of miR-9, miR-34 and miR-124 differs in *agn^ts3^* from that in *Berlin*, *Canton S* and Oregon-R (Savvateeva-Popova et al., 2017). miR-9 and miR-124 are also expressed in early development (0–12 hrs) (Sempere et al., 2003), miR-34 is detected in embryos till zygotic reduction (Soni et al., 2013). As known, the switch from maternal to zygotic development program occurs between the second and the third hours of embryonic stage, hence miRNA found in early development have maternal origin (Schier, 2007). These miRNAs targets are Swi/Snf-like complex, neural-progenitorspecific npBAF, repressor-element-1-silencing transcription factor (REST belonging to 1 class of histone deacetylases (HDAC1/2) and silent information regulator 1 (SIRT1) – 3 class of NAD+-dependent histone deacetylases involved in heterochromatin formation, Bourassa, Ratan, 2014). They are involved in neurogenesis, dendrite morphogenesis and axon guidance which depend on global chromatin remodeling.

Therefore, it is not surprising that in cross *agn^ts3^*×Berlin ectopic contacts between 11AB and regions containing genes involved in chromosome remodeling are formed with frequency characteristic for the maternal genome. The products of these genes are: Tip60 – histone acetyltransferase, HDAC6 – histone deacetylase; mxc – regulator of histone synthesis of Polycomb group; Top1 – DNA topoisomerase. However, only two regions containing genes HDAC6 and Top1 are present in cross Berlin×*agn^ts3^*.

Noteworthy, new knowledge about topologically associating domains (TADs) indicates that polytene, diploid, and embryonic TADs condensation along the chromosome axis is just the same everywhere (Eagen et al., 2015). Moreover, comparison of TADs with 3D chromatin organization revealed by the Hi-C method confirms that the interphase nucleus spatial organization into TADs is directly represented by banding pattern of polytene chromosomes (Kolesnikova, 2018). Therefore, this allows to bridge the ratio of genes forming ectopic contacts in a mode of either maternal, or paternal strain in reciprocal crosses and their physiologic manifestations. The later might result from alterations in the 11AB region architecture. As shown in Fig. 3, a, the *agn^ts3^*-like matroclinic mode of inheritance is pertinent to genes responsible for actin and microtubules-binding proteins with motor function and neurodevelopment. Noteworthy, the state of actin remodeling determining neurologic manifestations is a diagnostic feature of *agn^ts3^* (Savvateeva-Popova et al., 2017).

The regions with FECs similar in reciprocal hybrids, but differing from parents, i. e. manifesting hybrid properties, contain a large set of genes responsible for motor functions.

Figure 3, c shows genes and biological processes for chromosomal regions forming ectopic contacts with X:11AB only in hybrids or mainly in hybrids compared to parents. These processes are pertinent to main manifestations of *agn^ts3^*: meiosis, reparation, recombination; transcription factors; metabolism; proliferation; actin-binding proteins, microtubuleassociated proteins.

Interestingly, in the cross Berlin×*agn^ts3^* the chromosomal bands 8D, 12E, and 19D demonstrate FECs significantly exceeding these of parents. These bands contain genes involved in taste and odor perception and neurodevelopment, in particular of the mushroom bodies of the brain. The other examples of father strain manifestations might result from activities of trans-acting factors, such as miRNAs (Wittkopp et al., 2006).

miR-974 is involved in memory processes: its lowered expression impairs memory formation (Busto et al., 2015). Decrease in its content in olfactory neurons and the mushroom body V2 neurons promotes 3-hour memory. Noteworthy, the content of miR-974 is decreased both in *agn^ts3^* and in progeny of Berlin×*agn^ts3^* (impaired 3-hour memory) and is similar to wild type in the *agn^ts3^*×Berlin cross (normal memory). Likely, miR-974 might act as trans-acting factor presumed to regulate genes in patroclinic mode. The prevailing role of the paternal genome in memory formation is evident in *Canton S* and *agn^ts3^* reciprocal hybrids (Vasiljeva et al., 2019).

Taken together, our data indicate that the *agnostic* locus might belong to the class of quantitative trait loci (QTL) (Qin et al., 2019).

## Conclusion

One of the requirements of predictive and personalized medicine is consideration of POEs for prognosis of clinical phenotype of many multifactorial neuropsychiatric disorders. These different and individual manifestations of cognitive abilities and motor functions in patients with the same disease, i. e. behavioral plasticity, results from genome plasticity provoked by 3D chromatin architecture of the nerve cells nuclei. The evolutionary gene conservation approves the usage of simple low cost, fast and efficient models as Drosophila to probe the causes, consequences and mechanisms of pathology leading to human disease (Peffer et al., 2015). The Drosophila *agnostic* LIMK1 gene is a good candidate for linking the neuronal activity (spine remodeling, neurite outgrowth, trafficking of intracellular components, postsynaptic density functioning) and genetic apparatus (transcription machinery, chromatin-remodeling factors). Additionally, quite recent and unexpected findings (Davis, Zhong, 2017) reveal a new target of intellectual disabilities: learning acquisition and memory erasure (forgetting) are governed by different signal cascades, correspondently cAMP-dependent and actin remodeling cascade small GTPase Rac1 – LIMK1 (the key enzyme of actin remodeling LIM-kinase 1) and its phosphorylation substrate сofilin. The absence of Rac1-dependent forgetting causes the autistic spectrum disorders. Expression changes (active or non-active state) of LIMK1 and cofilin lead to different neurological disorders. Therefore, in the tradition of Russian genetic school (Lobashev et al., 1973), the *agnostic* gene might be a functional link between genetic and cytogenetic processes within the nervous system and serve as a model for elucidating both the maternal and paternal modes of transgenerational inheritance.

## Conflict of interest

The authors declare no conflict of interest.
